# Combination of esomeprazole and pirfenidone enhances antifibrotic efficacy in vitro and in a mouse model of TGFβ-induced lung fibrosis

**DOI:** 10.1038/s41598-022-24985-x

**Published:** 2022-11-30

**Authors:** Afshin Ebrahimpour, Manisha Ahir, Min Wang, Anil G. Jegga, Mark D. Bonnen, N. Tony Eissa, Sydney B. Montesi, Ganesh Raghu, Yohannes T. Ghebre

**Affiliations:** 1 Department of Radiation Oncology, Baylor College of Medicine, One Baylor Plaza, Houston, TX 77030 USA; 2 Division of Biomedical Informatics, Department of Pediatrics, Cincinnati Children’s Hospital Medical Center, University of Cincinnati College of Medicine, Cincinnati, OH 45229 USA; 3 Department of Radiation Oncology, The University of Texas Health Science Center at San Antonio, San Antonio, TX 78229 USA; 4 Department of Medicine, University of California, Irvine School of Medicine, Irvine, CA 92697 USA; 5 Division of Pulmonary and Critical Care Medicine, Massachusetts General Hospital, Boston, MA 02114 USA; 6 Division of Pulmonary and Critical Care Medicine, Center for Interstitial Lung Disease, University of Washington, Seattle, WA 98195 USA; 7 Department of Medicine, Section on Pulmonary and Critical Care Medicine, Baylor College of Medicine, Houston, TX 77030 USA; 8 Dan L. Duncan Comprehensive Cancer Center, Baylor College of Medicine, Houston, TX 77030 USA

**Keywords:** Animal disease models, Respiratory system models

## Abstract

Idiopathic pulmonary fibrosis (IPF) is a progressive and fatal lung disease of unknown etiology. Currently, pirfenidone and nintedanib are the only FDA-approved drugs for the treatment of IPF and are now the standard of care. This is a significant step in slowing down the progression of the disease. However, the drugs are unable to stop or reverse established fibrosis. Several retrospective clinical studies indicate that proton pump inhibitors (PPIs; FDA-approved to treat gastroesophageal reflux) are associated with favorable outcomes in patients with IPF, and emerging preclinical studies report that PPIs possess antifibrotic activity. In this study, we evaluated the antifibrotic efficacy of the PPI esomeprazole when combined with pirfenidone in vitro and in vivo. In cell culture studies of IPF lung fibroblasts, we assessed the effect of the combination on several fibrosis-related biological processes including TGFβ-induced cell proliferation, cell migration, cell contraction, and collagen production. In an in vivo study, we used mouse model of TGFβ-induced lung fibrosis to evaluate the antifibrotic efficacy of esomeprazole/pirfenidone combination. We also performed computational studies to understand the molecular mechanisms by which esomeprazole and/or pirfenidone regulate lung fibrosis. We found that esomeprazole significantly enhanced the anti-proliferative effect of pirfenidone and favorably modulated TGFβ-induced cell migration and contraction of collagen gels. We also found that the combination significantly suppressed collagen production in response to TGFβ in comparison to pirfenidone monotherapy. In addition, our animal study demonstrated that the combination therapy effectively inhibited the differentiation of lung fibroblasts into alpha smooth muscle actin (αSMA)-expressing myofibroblasts to attenuate the progression of lung fibrosis. Finally, our bioinformatics study of cells treated with esomeprazole or pirfenidone revealed that the drugs target several extracellular matrix (ECM) related pathways with esomeprazole preferentially targeting collagen family members while pirfenidone targets the keratins. In conclusion, our cell biological, computational, and in vivo studies show that the PPI esomeprazole enhances the antifibrotic efficacy of pirfenidone through complementary molecular mechanisms. This data supports the initiation of prospective clinical studies aimed at repurposing PPIs for the treatment of IPF and other fibrotic lung diseases where pirfenidone is prescribed.

## Introduction

IPF is a deadly orphan disease of unknown etiology that causes progressive loss of lung function. The lungs of IPF patients are characterized by distortion of airway structures including the alveoli, interstitium, airspaces and the vasculature resulting in dyspnea and death within an average of 4 years from the time of diagnosis^1^. Current understanding of the disease process indicates that repetitive lung injury in susceptible individuals promotes uncontrolled proliferation of fibroblasts and differentiation into collagen-synthesizing myofibroblasts. Histologically, the areas of injury and active fibrosis are characterized by interstitial scarring, sub-pleural honeycombing and fibroblastic foci. In the US, the disease has an incidence of 93.7 cases per 100,000 in these 65 years of age and older, and includes over 125,000 cases^1^.

Following the FDA approval of pirfenidone and nintedanib for the treatment of IPF in late 2014, the two drugs have been in clinical use globally and are now considered the standard of care all over the world. Although this is an important milestone in the treatment for IPF, these drugs only slow the progression of the disease but are unable to stop or reverse established fibrosis^2,3^. Nonetheless, pirfenidone and nintedanib have profoundly impacted the way translational and clinical studies are designed and conducted in IPF. Such emerging studies have to account for standard of care treatment with either pirfenidone or nintedanib and need to be designed in the context of these antifibrotic therapies. In this regard, it is important to test and develop new compounds that mechanistically target signaling pathways that play pathobiologic role in IPF but are not targeted by the current drugs. Such compounds have the potential to be combined with the existing antifibrotic drugs for enhanced efficacy. According to the studies that our group has conducted, proton pump inhibitors (FDA-approved to treat gastroesophageal reflux) might be combined with pirfenidone for enhanced antifibrotic activity.

Since their FDA approval in the late 1980s, PPIs such as omeprazole (Prilosec), lansoprazole (Prevacid), dexlansoprazole (Dexilant), rabeprazole (Aciphex), pantoprazole (Protonix) and esomeprazole (Nexium) have been widely used in clinical practice to manage patients with gastrointestinal disorders including gastroesophageal reflux (GER) disease (GERD), Barrett’s esophagus and Zollinger-Ellison (ZE) Syndrome. The approved oral dose of PPI varies from 15 to 60 mg once or twice a day. At these doses, the plasma drug concentration ranges from 10 to 25 µM^4–7^. However, significantly higher than the approved doses of PPI (up to 360 mg) have been safely used in patients with ZE Syndrome and cancer to achieve close to 100 µM plasma concentrations^6,8–10^.

Emerging preclinical studies indicate that PPIs modulate several pro-inflammatory cytokines to control tissue inflammation^11–14^. Additional studies indicate that PPIs simultaneously inhibit profibrotic molecules and induce antifibrotic mechanisms to control tissue fibrosis^15–17^. Several retrospective clinical studies or post-hoc analysis of data from IPF clinical trials also support the association of PPI use and favorable outcomes in patients with well-defined IPF. Some of the beneficial outcomes associated with the use of PPIs include stabilized or improved lung function, reduced episodes of acute exacerbations, slower loss of lung function, lower IPF-related mortality and lower radiologic fibrosis score^15,18–22^. For example, in the study reported by Kreuter et al., the combination of antacids (with PPIs representing over 90% of the medications compared to other class of antacids) with pirfenidone decreased IPF-related mortality, death or 6-min walk distance (6MWD), all-cause mortality, progression-free survival, and favored significant preservation of lung function compared to pirfenidone alone in an unadjusted analyses in participants enrolled in the CAPACITY or ASCEND trials^22^. These results combined with our prior preclinical data supporting an antifibrotic effect of PPIs encouraged us to evaluate the combination of pirfenidone with the PPI esomeprazole in controlling biological processes that drive lung fibrosis. For this, we used cell biological, computational and animal models to understand how PPIs may contribute to enhanced antifibrotic efficacy in combination with pirfenidone.

## Materials and methods

### Proliferation of IPF lung fibroblasts treated with esomeprazole/pirfenidone combination

IPF lung fibroblasts were expanded on 75 cm^2^ (“T75”) flasks and batch-frozen in liquid nitrogen for downstream experiments. Subsequently, 3.5 × 10^3^ cells were seeded in a 96-well plate and cultured overnight to allow adherence to the plate. The next day, the cells were synchronized by serum starvation prior to stimulating proliferation using recombinant human TGFβ (10 ng/mL; Peprotech; cat # 100-21) for 24 h. Thereafter, the cells were treated with vehicle (dH_2_O), esomeprazole (100 μM), pirfenidone (1 mM), or a combination of esomeprazole (100 μM) and pirfenidone (1 mM) for another 24 h prior to adding 5-bromo-2-deoxyuridine (BrdU; 1:500) (Sigma, cat # 2750) overnight to monitor cell proliferation under the various treatment conditions. Cell proliferation was determined from the incorporation of BrdU into the DNA of the dividing cells by measuring absorbance (OD 450 nm) spectrophotometrically using Tecan Spark 20 M plate reader. Finally, the proliferation data was compared among the groups to assess the effect of esomeprazole/pirfenidone combination compared to either treatment alone.

### Esomeprazole/pirfenidone combination on TGFβ-induced migration of IPF lung fibroblasts

To assess the effect of esomeprazole and its combination with pirfenidone on the expansion and migration of IPF lung fibroblasts in response to TGFβ, the CytoSelect™ 24-Well Wound Healing Assay (Cell Biolabs; cat # CBA-120) was used. First, the provided inserts were placed inside the wells and 1 × 10^6^ cells were seeded in 250 µL fully-supplemented DMEM. The cells were allowed to form a monolayer around the insert for 24 h. The next day, the cells were synchronized by serum starvation prior to removing the inserts to create a 0.9 mm × 1.8 mm scratch area in each of the wells. The cells were then washed with PBS and imaged for baseline scratch area measurement prior to treatment with vehicle, esomeprazole (100 µM), pirfenidone (1 mM) or the combination for up to 72 h in the absence or presence of TGFβ (10 ng/mL) in triplicates. Finally, images were captured using bright-field microscopy (Leica Microsystems, Germany) to determine the effect of esomeprazole and/or pirfenidone on the migration of IPF lung fibroblasts in response to the mitogenic cytokine TGFβ. The remaining scratch area after treatment was measured using a scaled ruler and was converted into percentage of scratch closure for comparison.

### Esomeprazole/pirfenidone combination on the contractility of IPF lung fibroblasts

One of the characteristics of TGFβ-treated fibroblasts or bona fide myofibroblasts is increased contractility of collagen matrices. In vitro, this is assessed by a Cell Contraction Assay. To evaluate the effect of combining the antifibrotic drug pirfenidone with esomeprazole on the contractility of TGFβ-treated IPF lung fibroblasts, we used Cell Biolabs’ Cell Contraction Assay (cat # CBA-201) and the protocol provided in the kit. First, Collagen Gel Working Solution was prepared by transferring 9.54 mL of the provided Collagen Solution into a cold sterile tube and mixing with 5× DMEM and 340 µL Neutralization Solution. The Solution was mixed well, and the resulting Collagen Gel Working Solution was kept on ice for subsequent use. Next, 2 × 10^6^ IPF lung fibroblasts were resuspended in DMEM and mixed with the Collagen Gel Working Solution in a 1:4 ratio. Subsequently, 500 µL of the cell-collagen mixture was added in each well of a 24-well plate and incubated at 37 °C for 1 h to allow polymerization of the collagen. Next, 1 mL of DMEM was added on top of the collagen gel lattice and the plate was incubated for 48 h to allow development of cellular stress fibers. Subsequently, the cells were treated with vehicle, esomeprazole (100 µM), pirfenidone (1 mM) or the combination thereof for up to 48 h in the absence or presence of TGFβ (10 ng/mL). Finally, cell contraction was initiated by releasing the collagen gels from the walls of the plate with a sterile spatula, and the change in collagen gel size (i.e., contraction index) was measured with a ruler at 24- and 48- hours post-treatment for comparison.

### The effect of esomeprazole/pirfenidone combination on collagen production by IPF lung fibroblasts

To evaluate the effect of combining esomeprazole and pirfenidone on the production of collagen by IPF lung fibroblasts, we used the colorimetric Sircol assay kit (BioColor; cat # S1000) and the provided experimental protocol. First, collagen was isolated and concentrated by transferring 1 mL of conditioned media from vehicle, esomeprazole (100 µM), pirfenidone (1 mM) or the combination of esomeprazole and pirfenidone treated IPF lung fibroblasts (with or without TGFβ) into low protein binding 1.5 mL microcentrifuge tubes and adding 200 µL of cold Isolation and Concentration Reagent. The tubes were mixed by gentle vortexing and incubated overnight in a container containing ice-water mix. The next day, the tubes were centrifuged at 12,000 rpm for 10 min and 1000 µL of supernatant was removed from each tube prior to adding the same volume of Sircol Dye Reagent. The samples were placed in a mechanical shaker and incubated for 30 min at room temperature to allow the formation of collagen-dye complex. In parallel, fresh DMEM was prepared as a blank control and the provided collagen solution was used to prepare standards. Following the incubation, the samples were centrifuged at 12,000 rpm for 10 min and the tubes were carefully inverted to drain the contents (i.e., unbound dye) and access the firmly packed collagen-dye complex at the bottom of the tubes. Subsequently, 750 µL of ice-cold Acid-Salt Wash Reagent was added to each of the sample tubes and centrifuged as above to remove any remaining unbound dye. To release and recover the collagen bound dye, 250 µL of Alkali Reagent was added to the blanks, standards and samples. The collagen-bound dye was released into solution by vortexing each of the tubes for 5 min. Finally, 200 µL of each sample was transferred to individual wells of a 96-well plate and absorbance, proportional to the intensity of collagen, was measured at 555 nm. The concentration of collagen was obtained from the standard curve for comparison.

### Computational analysis

Gene expression profiles of A549 human lung epithelial cells treated with thousands of small molecules including FDA-approved drugs such as esomeprazole and pirfenidone is deposited in a publicly-accessible database known as the Library of Integrated Network-based Cellular Signatures (LINCS)^23^. We queried IPF lung transcriptome (GSE53845)^24^ against the gene expression profiles generated from esomeprazole or pirfenidone treated cells from the LINCS database using the Connectivity Map approach^25^. Finally, genes that were reciprocally regulated (i.e., up in IPF but down in esomeprazole or pirfenidone treatment and vice versa) were subjected to functional enrichment analysis using ToppFun^26^ to identify enriched biological processes and pathways. Results from the enrichment analyses were visualized using Cytoscape software^27^.

### Antifibrotic efficacy of esomeprazole/pirfenidone combination in a mouse model of TGFβ-induced lung fibrosis

We used 18 months old male C57BL/6J mice (n = 10–12/group) to induce TGFβ-mediated lung fibrosis and assess the efficacy of esomeprazole/pirfenidone combination in regulating the fibrosis. First, hair was removed from the trachea area and total body weight was measured prior to randomization of the animals into sham control (group 1; n = 10), vehicle control (group 2; n = 12), esomeprazole (group 3; n = 12), pirfenidone (group 4; n = 12), or esomeprazole/pirfenidone combination (group 5; n = 12). Next, the animals were anesthetized with a combination of ketamine (80 mg/kg) and xylazine (16 mg/kg) intraperitoneally to achieve a deep plane of anesthesia. Subsequently, adenovirus encoding for TGFβ (6 × 10^6^ pfu; Vector Biolabs; cat # ADV-274099) was intratracheally (IT) administered in the animals randomized to groups 2–5. For group 1, the animals received adenoviral vector without TGFβ (day 0). Subsequently, all the animals were allowed to recover, and starting from day 10, the animals in groups 1 and 2 were treated with vehicle (water). The animals in groups 3–5 were treated with esomeprazole (30 mg/kg), pirfenidone (100 mg/kg), and esomeprazole/pirfenidone combination, respectively. All the treatments were administered daily by oral gavage, and for the animals in group 5, the same dose of esomeprazole and pirfenidone used to treat the animals in groups 3 and 4 was used. Total body weight was monitored every week and all the animals were euthanized according to an IACUC-approved “Euthanasia in Rodents” policy with an overdose of ketamine and xylazine followed by bilateral opening of the thorax 28 days after the TGFβ challenge. Organ weight (lungs, heart, liver, kidneys) were measured to assess gross toxicity, and the left lungs were fixed in 10% paraformaldehyde for histopathological studies including Masson’s trichrome stain for collagen and αSMA immunohistochemistry. The right lungs from 50% of the animals were homogenized for tissue collagen content using the Sircol assay described above. For the histopathological studies, the degree of fibrosis was scored on a scale of 1–4 where 1 = minimal; 2 = mild; 3 = moderate; and 4 = severe as we described^15^. The expression of αSMA was graded on a scale of 1+ to 3+ where 1+ = minimal staining; 2+ = moderate staining; and 3+ = intense staining.

### Statistical analysis

The number of animals per study group needed was calculated using Power and Sample Size calculation (PS v3.1.2; Vanderbilt University) to at least detect a difference in means (δ) of 0.25 with an estimated standard deviation (σ) of 0.2 at significance (α) of 0.05 with 80% power (β). In a previous study of esomeprazole monotherapy, the antifibrotic response was normally distributed when a similar statistical test was performed^15^. All in vitro assays were run in triplicates and were repeated at least three times to ensure reproducibility. All the data was analyzed using one-way ANOVA followed by Bonferroni post-hoc test (GraphPad prism) and was expressed as mean ± SEM. Differences are considered statistically significant at p value below 0.05 (p < 0.05).

### Animal study approval

Baylor College of Medicine Animal Care and Use Committee approved the animal study described in the paper. The study was carried out in compliance with Baylor College of Medicine Guidelines, which are based on the National Institutes of Health’s Guide for the Care and Use of Laboratory Animals, and with the ARRIVE guidelines. Lung fibroblasts isolated from IPF patients were purchased from a commercial source (Lonza; Walkersville, MD).

## Results

### Esomeprazole enhances the antiproliferative effect of pirfenidone on lung fibroblasts

**Figure 1:**
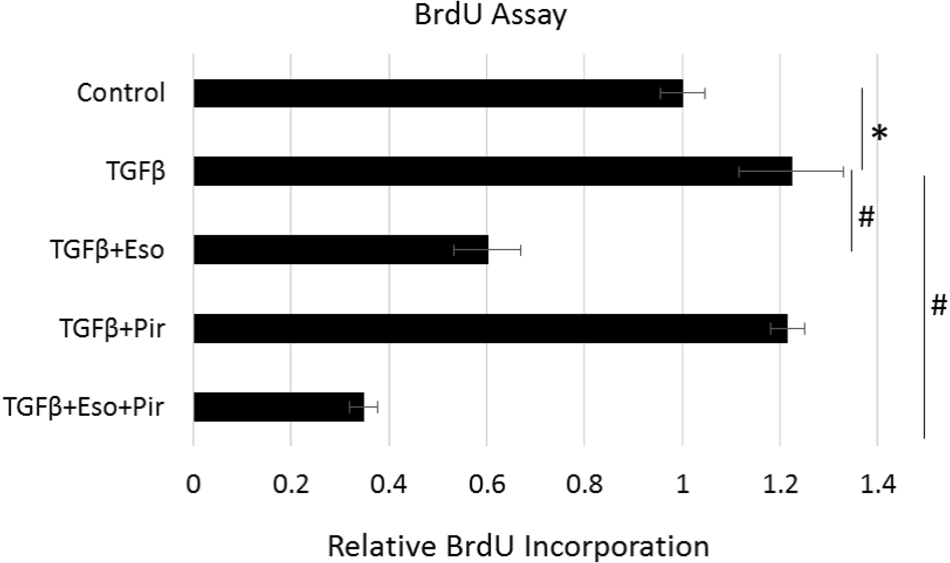
Bromodeoxyuridine (BrdU) assay showing anti-proliferative effects of esomeprazole, pirfenidone, and the combination of both drugs. Fibroblasts derived from the lungs of IPF patients were stimulated with TGFβ (10 ng/mL) for 24 h and treated with vehicle (dH_2_O), esomeprazole (100 µM), pirfenidone (1 mM) or esomeprazole (100 µM) and pirfenidone (1 mM) combination for another 24 h. Cell proliferation, proportional to the incorporation of BrdU, was assessed spectrophotometrically by measuring absorbance at 450 nm. Data is mean ± SEM from triplicate experiments and represents three independent experiments. *p < 0.05 compared to no TGFβ control; ^#^p < 0.05 compared to TGFβ only control.

Over-proliferation of lung fibroblasts and differentiation into myofibroblasts are believed to form the fibroblastic foci implicated in driving the progression of pulmonary fibrosis^28,29^. High doses of pirfenidone have been reported to modulate the proliferation of human lung fibroblasts in response to TGFβ^30^. Intriguingly, combination of pirfenidone with esomeprazole significantly enhanced the anti-proliferative effect (Fig. 1). More specifically, the BrdU assay data shows that TGFβ significantly enhanced the proliferation of IPF lung fibroblasts compared to untreated controls. By contrast, esomeprazole significantly inhibited proliferation of the cells in response to TGFβ. The anti-proliferative effect was further enhanced when esomeprazole was combined with pirfenidone (Fig. 1).

### TGFβ-induced migration of IPF lung fibroblasts is blocked by the combination of esomeprazole and pirfenidone

**Figure 2:**
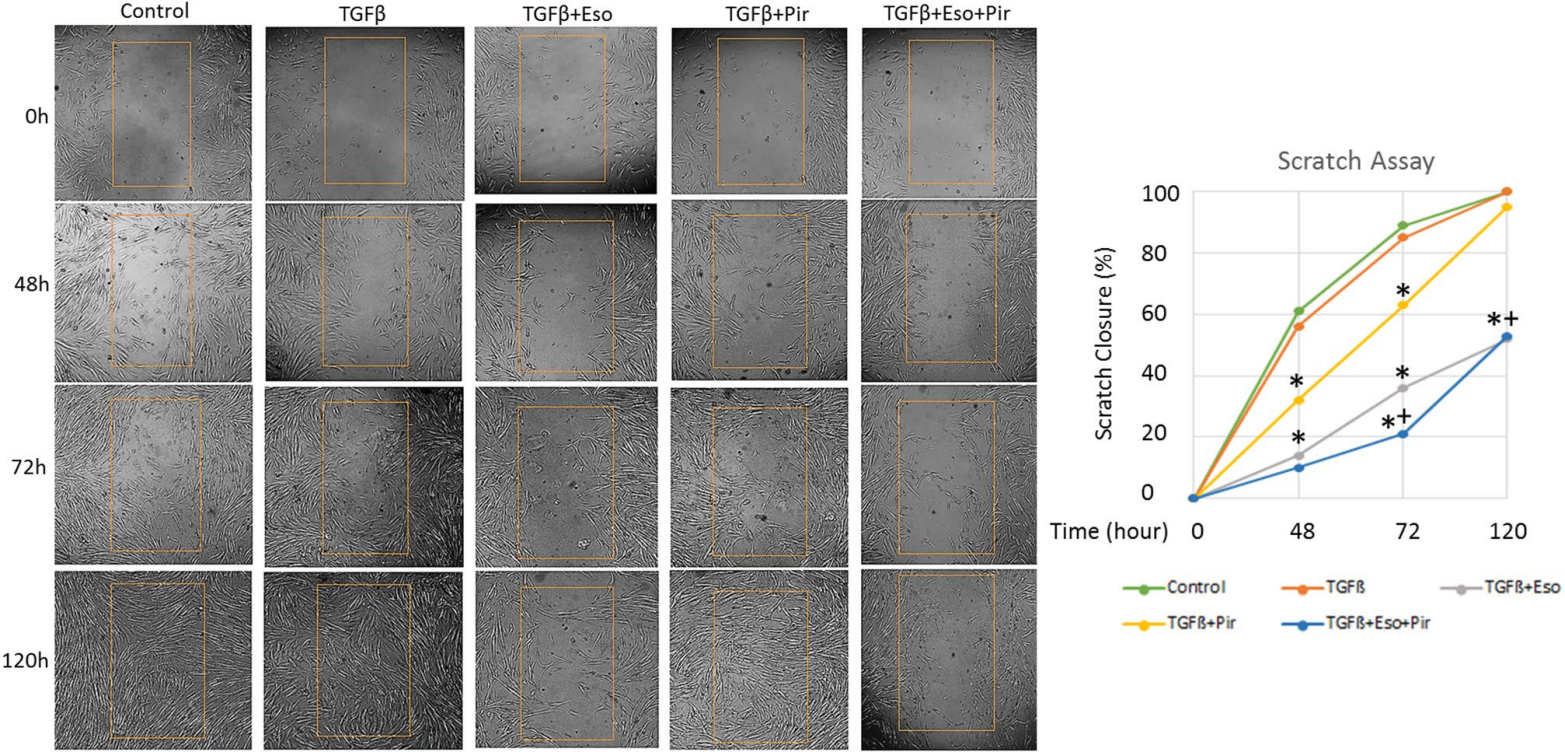
Cell migration assay showing the effect of esomeprazole (Eso), pirfenidone (Pir), and the combination on cell migration. Monolayers of human IPF lung fibroblasts were denuded to create cell-free area prior to stimulation with TGFβ (10 ng/mL) for 48, 72 or 120 h in the absence or presence of esomeprazole (100 µM), pirfenidone (1 mM) or esomeprazole (100 µM) and pirfenidone (1 mM) combination. Cell migration to close the denuded area, shown as averaged percentage of scratch closure, was quantified for comparison. Data represents three independent experiments. *p < 0.05 vs TGFβ only control and ^+^p < 0.05 vs pirfenidone alone group.

TGFβ is a known mitogen and stimulator of fibroblast migration in scratch assays that denude monolayer of cells to study cell migration in response to treatment with various bioactive molecules^31^. In our study, treatment with TGFβ progressively enhanced the migration of IPF lung fibroblasts into the denuded area resulting in complete closure of the area within 120 h (Fig. 2). As expected, treatment with pirfenidone transiently inhibited the cell migration, showing about 40% inhibition by 72 h. However, this inhibition was mostly reversed at 96 h post-treatment. By contrast, esomeprazole sustainably inhibited the migration of IPF lung fibroblasts in response to TGFβ with the significance of inhibition holding up for at least 120 h after treatment. The combination of esomeprazole with pirfenidone was more effective in inhibiting cell migration than either treatment alone. The combination treatment inhibited the migration of IPF lung fibroblasts by about 90% at 48 h post-treatment (Fig. 2).

### The contractility of IPF lung fibroblasts is attenuated by esomeprazole and pirfenidone combination

**Figure 3:**
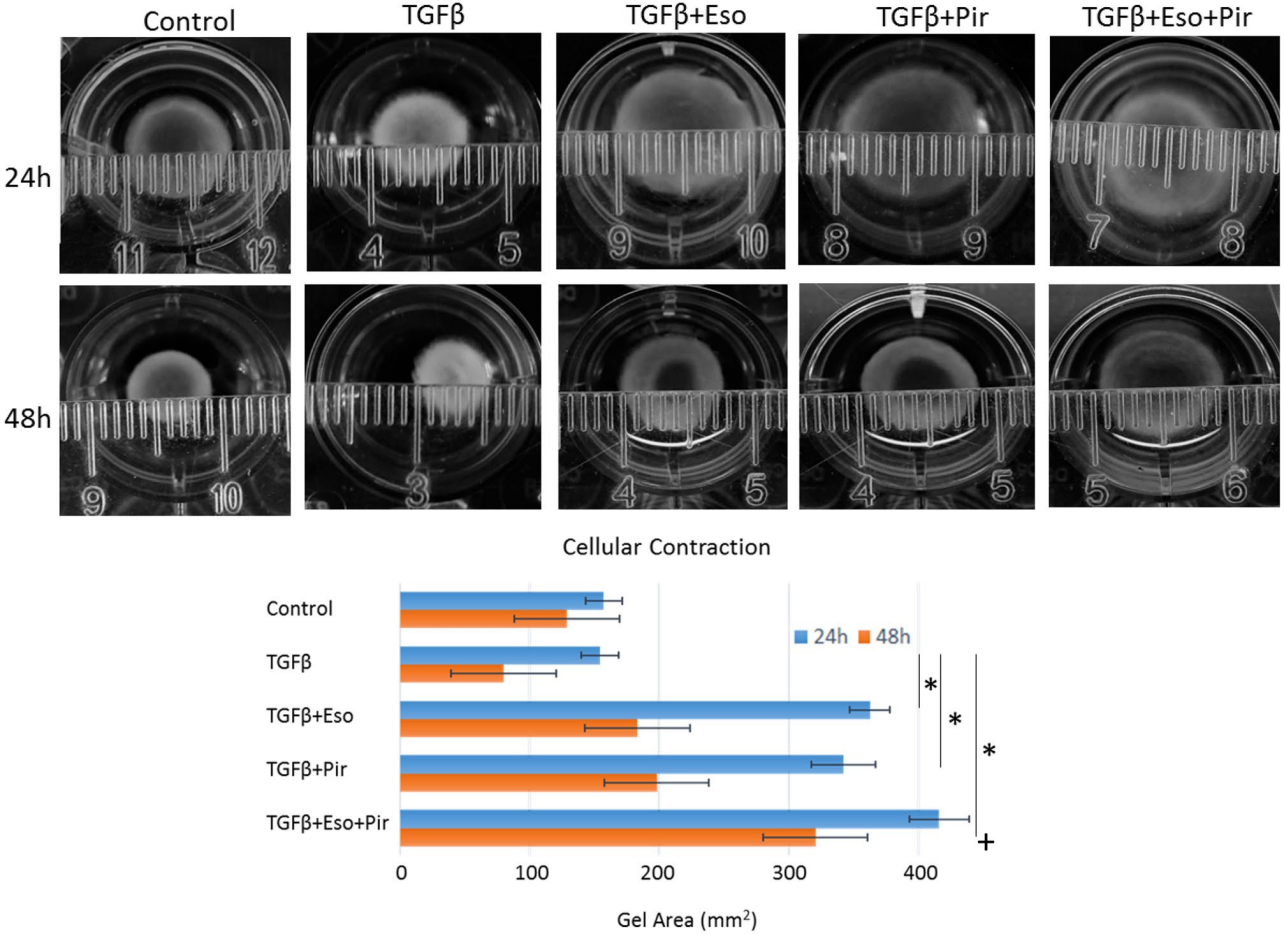
The effect of esomeprazole (Eso), pirfenidone (Pir) or the combination on the contractility of IPF lung fibroblasts using cell contraction assay. The cells were treated with TGFβ (10 ng/mL) for 24 or 48 h in the absence or presence of esomeprazole (100 µM), pirfenidone (1 mM) or esomeprazole (100 µM) and pirfenidone (1 mM) combination. The contractility of cells embedded in collagen gels was followed over time and the area of the gel was calculated for comparison. Data represents three independent experiments. *p < 0.05 vs TGFβ only control and ^+^p < 0.05 vs pirfenidone alone group.

Contraction of collagen gels in response to TGFβ stimulation is a classic characteristics of myofibroblasts^32,33^. This characteristic is believed to increase contraction forces such as tensile strength to promote matrix stiffness in vitro and in vivo. As expected, we found that TGFβ treatment of IPF lung fibroblasts embedded in collagen gels increased the contractility of the gels in a time-dependent manner (Fig. 3). Not surprisingly, treatment with pirfenidone augmented the effect of TGFβ and significantly attenuated the contractile property of the cells with a peak inhibition at 24 h post-treatment. Similarly, treatment with esomeprazole inhibited the contractility of IPF lung fibroblasts stimulated with TGFβ. Combination of esomeprazole and pirfenidone efficiently blocked the contractile force exerted against the collagen lattice to negate changes in the diameter of the gels for up to 48 h post-treatment (Fig. 3).

### The combination of esomeprazole and pirfenidone suppresses TGFβ-induced collagen production by IPF lung fibroblasts

**Figure 4:**
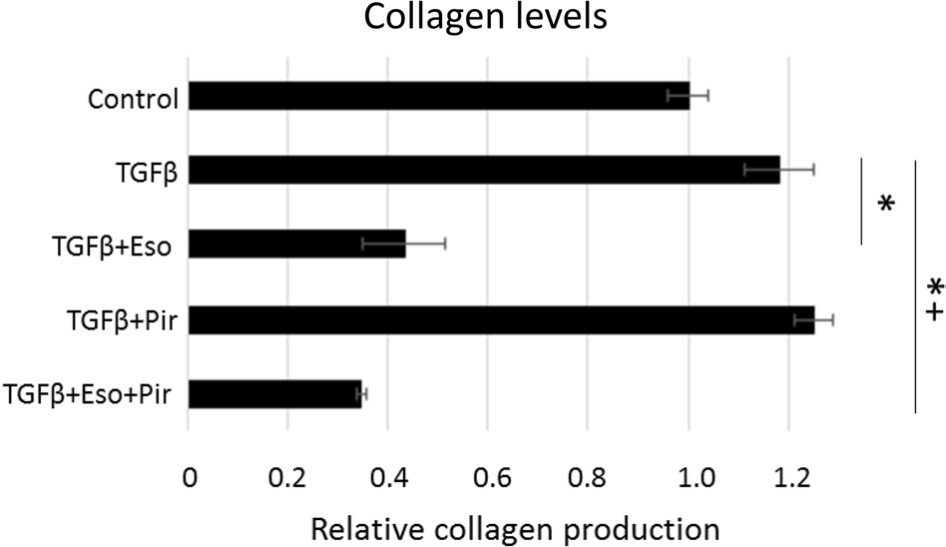
Sircol assay data showing the effect of esomeprazole, pirfenidone or the combination on the production of soluble collagen. Human IPF lung fibroblasts were treated with TGFβ (10 ng/mL) in the absence or presence of esomeprazole (100 µM), pirfenidone (1 mM) or esomeprazole (100 µM) and pirfenidone (1 mM) combination for 24 h. The amount of collagen, proportional to the intensity of red color, was assessed spectrophotometrically by measuring absorbance at 555 nm. Data is Mean ± SEM from triplicate experiments and represents three independent experiments. *p < 0.05 compared to TGFβ only control and ^+^p < 0.05 vs pirfenidone alone group.

Overproduction of collagen and other ECM proteins by TGFβ-stimulated lung fibroblasts is a hallmark of pulmonary fibrosis including IPF^34,35^. In this study, we found that stimulation of IPF lung fibroblasts with TGFβ significantly enhanced the release of soluble collagen into the conditioned media (Fig. 4). By contrast, treatment with esomeprazole alone attenuated collagen production by about 60%. Although pirfenidone alone was not effective in reducing collagen levels in TGFβ-treated IPF lung fibroblasts, the combination of both esomeprazole and pirfenidone attenuated collagen production by over 60% (Fig. 4).

### Esomeprazole and pirfenidone favorably regulate several fibrosis-related gene networks

**Figure 5:**
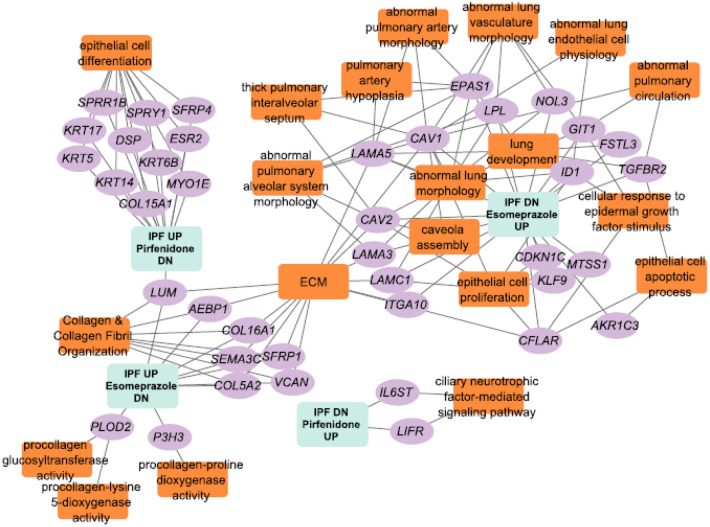
Network representation of genes and biological processes and pathways that are dysregulated in IPF and are controlled by esomeprazole, pirfenidone, or the combination. Orange-colored rectangles represent enriched biological processes, pathways or phenotypes while the purple-colored oval shapes represent genes that are up or down regulated by esomeprazole, pirfenidone or the combination but reciprocally regulated in IPF. The gene expression data for esomeprazole and pirfenidone treatment is from the Library of Integrated Network-based Cellular Signatures (LINCS) database while the IPF dysregulated gene expression data is from human patients. Functional enrichment analysis is done using ToppFun application and network generation is done using Cytoscape software.

We investigated the molecular mechanisms by which pirfenidone and esomeprazole regulate the biological processes that are associated with the development and/or progression of lung fibrosis. Querying of the LINCS database and comparison of differentially expressed genes (DEGs) from the two compounds revealed that there is some overlap, but they mostly regulate different genes or pathways (Fig. 5). Enrichment analysis of genes up/down regulated in the settings of esomeprazole or pirfenidone exposure using Kaminski^36^ and Banovich^37^ IPF single-cell atlas indicates that esomeprazole targets stromal cells while pirfenidone targets epithelial cells (Supplementary Table S1). Comparison of DEGs from these compounds with DEGs from IPF^24^ demonstrated that pirfenidone mainly targets the keratins while esomeprazole primarily targets collagens (Supplementary Table S1). Interestingly, combination of pirfenidone and esomeprazole significantly downregulated the expression of the profibrotic molecule lumican (Fig. 5 and Supplementary Table S1). Finally, functional enrichment analysis of the genes that are reciprocally regulated (i.e., upregulated in IPF but downregulated by pirfenidone or esomeprazole and vice versa) showed that several lung development and IPF-related pathways are favorably co-regulated by the two compounds (Fig. 5 and Supplementary Table S1).

### In vivo combination of esomeprazole and pirfenidone inhibits lung fibrosis more than either treatment alone

**Figure 6:**
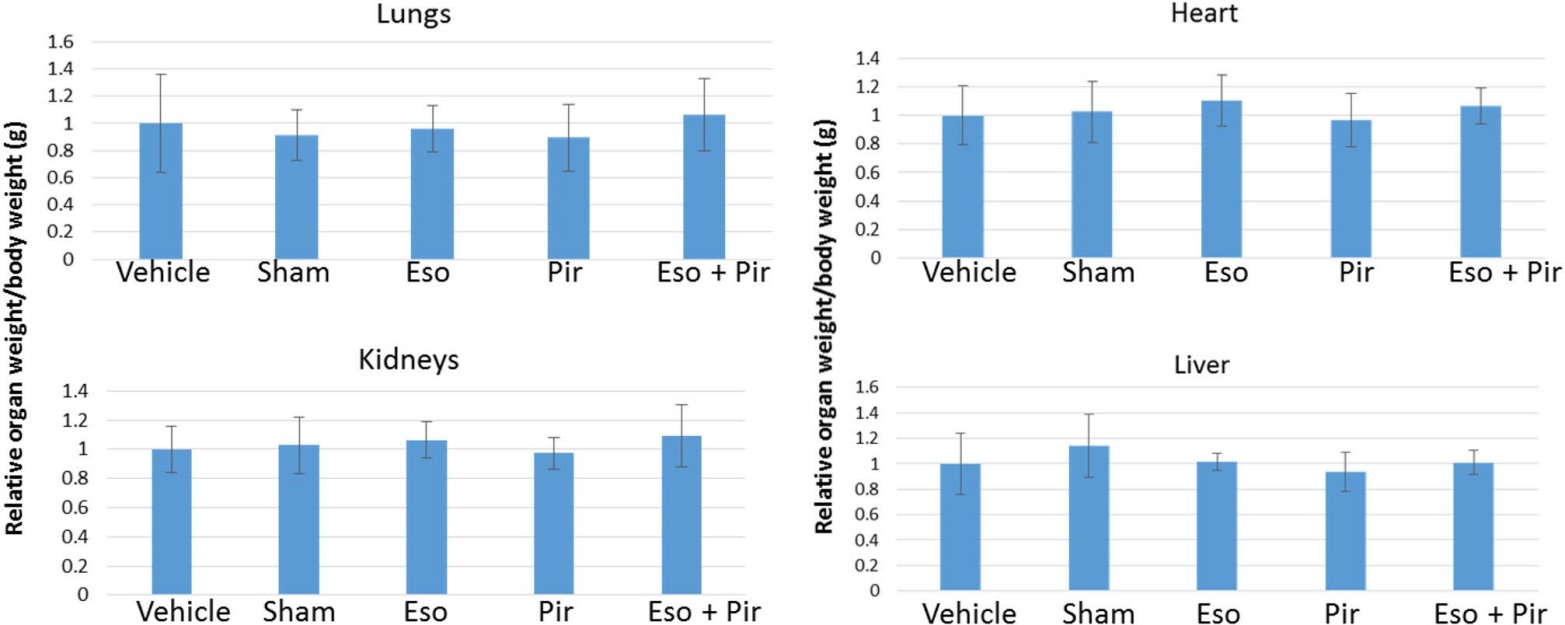
Measurement of organ weight in a TGFβ-induced lung fibrosis mouse model. The weight of the lungs, heart, liver and kidneys were normalized to the total body weight of the respective animal on the day of necropsy and was recorded for comparison. The weight of the kidneys and the lungs represent the total weight for the left and right organs. Combination of esomeprazole and pirfenidone did not significantly change the weight of any of the organs. Data is mean ± SEM from 10 to 12 animals per group.

**Figure 7:**
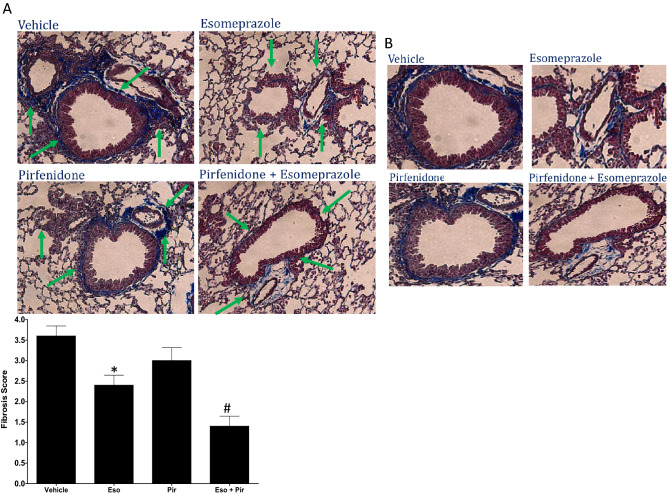
(**A**) Masson’s trichrome stain demonstrating antifibrotic effect of esomeprazole, pirfenidone or the combination. C57BL/6J mice were intratracheally challenged with adenoviral vector encoding TGFβ (6 × 10^6^ pfu) and orally treated with vehicle (dH_2_O), esomeprazole (Eso; 30 mg/kg), pirfenidone (Pir; 100 mg/kg) or esomeprazole (30 mg/kg) and pirfenidone (100 mg/kg) combination (Eso + Pir) starting from 10 days post-challenge. The animals were treated daily until the day of necropsy (day 28). The combination therapy effectively suppressed lung fibrosis (shown as blue stain) compared to esomeprazole or pirfenidone monotherapy (arrows). In panel (**B**), zoomed out images of the tissue included in the analysis are shown. The fibrosis scores are shown as a bar graph. Data is from 5 animals/group. *p < 0.05 compared to the vehicle group and ^#^p < 0.05 compared to pirfenidone alone.

**Figure 8:**
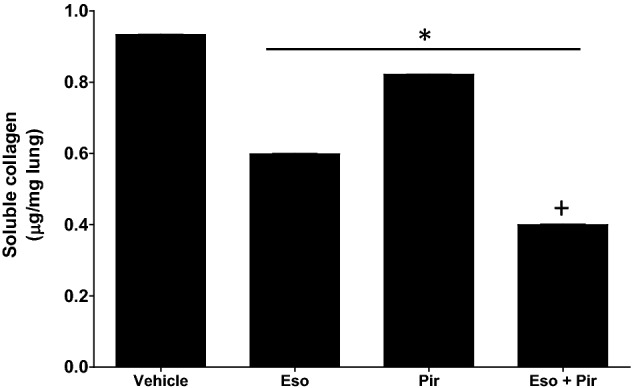
Measurement of collagen in lung homogenates from TGFβ-induced lung fibrosis mouse model. The right lungs of vehicle, esomeprazole, pirfenidone, and esomeprazole/pirfenidone combination were homogenized and analyzed for tissue collagen content by colorimetric assay. The homogenates from the right lungs were pooled together for each group (n = 5 animals/group) and assayed using Sircol assay following the manufacturer’s protocol. The amount of collagen was estimated from standard curves (OD = 555 nm) and was expressed as µg collagen per mg of wet tissue. Data is mean ± SEM from triplicate experiments. *p < 0.05 compared to TGFβ only control and ^+^p < 0.05 vs pirfenidone alone group.

**Figure 9:**
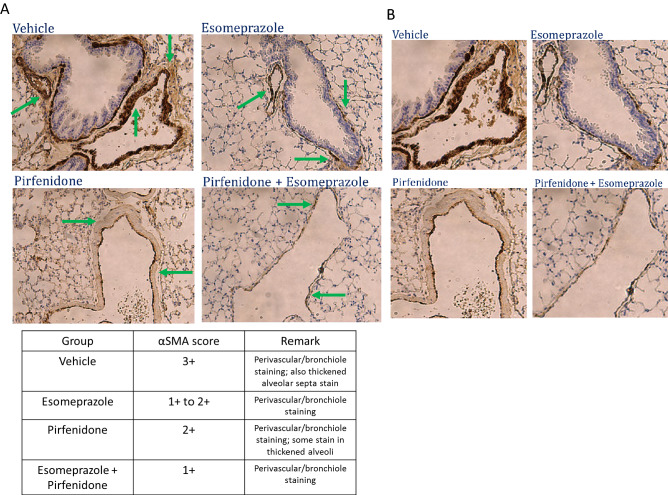
(**A**) Immunohistochemistry data showing alpha smooth muscle actin (αSMA) expression in a mouse model of TGFβ-induced lung fibrosis. The animals were challenged with adenoviral vector encoding TGFβ (6 × 10^6^ pfu) and orally treated with vehicle (dH_2_O), esomeprazole (30 mg/kg), pirfenidone (100 mg/kg) or esomeprazole (30 mg/kg) and pirfenidone (100 mg/kg) combination as described in the “Materials and methods” section. The data shows that the combination therapy inhibits lung tissue muscularization, as shown by reduced αSMA expression (brown stain), more effectively that the monotherapy (arrows). In panel (**B**), zoomed out images of the tissue included in the analysis are shown. The semi-quantitative scores are shown in the table and the data is representative of 5 animals/group.

Given the enhanced antifibrotic efficacy observed when pirfenidone was combined with esomeprazole in vitro, we evaluated the efficacy of the combination in vivo in a mouse model of TGFβ-induced lung fibrosis. We found that the combination drug was well-tolerated without gross toxicity to vital organs such as the lungs, heart, kidneys and liver as confirmed by lack of change in the weight of these organs normalized to the body weight at the time of euthanasia (Fig. 6). Masson’s trichrome stain showed that esomeprazole or pirfenidone monotherapy is effective in reducing lung fibrosis despite the drugs being administered 10 days after the induction of lung injury (Fig. 7). Intriguingly, the combination of esomeprazole and pirfenidone reduced lung fibrosis to a greater extent than either treatment alone (Figs. 7, 8). Consistently, the combination of esomeprazole and pirfenidone downregulated the expression of the myofibroblast marker αSMA more than monotherapy with esomeprazole or pirfenidone (Fig. 9). Notably, αSMA staining was more intense in areas of increased cellularity and fibrosis in the TGFβ only control. However, treatment with monotherapy or the combination therapy limited the staining to focal areas of fibrosis.

## Discussion

### Esomeprazole favorably regulates biological processes related to lung fibrosis to enhance the antifibrotic effect of pirfenidone

It is reported that 87–94% of IPF patients have abnormal acid gastroesophageal reflux (GER) or GER disease (GERD)^38,39^. As a result, many of these patients are prescribed antacids including PPIs such as esomeprazole to alleviate symptoms of GERD such as chest pain and heartburn. However, it is not known how the PPIs interact with standard of care drugs for IPF including pirfenidone. Data from three large prospective clinical trials (CAPACITY 004, 006 and ASCEND) that primarily evaluated the efficacy of pirfenidone in IPF revealed that IPF patients treated with pirfenidone and antacids (of whom ≥ 90% were on PPIs) had numerically favorable outcomes in progression-free survival, death or six minute walk distance (6MWD) decline by ≥ 10%, IPF-related mortality, and all-cause mortality^22^. Intriguingly, the study also reported that the combination of antacids and pirfenidone significantly slowed the decline in lung function (i.e., FVC decline ≥ 10%). Paradoxically, there is no clinical study that prospectively evaluated the combination of antacids such as esomeprazole and pirfenidone in patients with IPF.

Pre-clinical studies from our group reported that PPIs possess antifibrotic activities including regulation of fibroblast proliferation and suppression of lung fibrosis induced by bleomycin or smoke^15,16,40^. Recently, we reported that esomeprazole favorably regulates a network of genes involved in lung remodeling^41^. However, it is not known whether esomeprazole enhances the antifibrotic efficacy of pirfenidone or whether the two drugs overlap in the molecular mechanisms by which they control processes involved in lung fibrosis. Our present study demonstrates that the combination of esomeprazole and pirfenidone enhances the regulation of several biological processes that drive lung fibrosis including TGFβ-induced proliferation and migration of lung fibroblasts, contractility of collagen gels, as well as production of soluble collagen by TGFβ-stimulated IPF lung fibroblasts in vitro (Figs. 1, 2, 3, 4). In addition, our in vivo study demonstrated that the combination of esomeprazole and pirfenidone is more effective in suppressing lung fibrosis than esomeprazole or pirfenidone monotherapy as shown by reduction in fibrosis and αSMA expression (Figs. 7, 8, 9). Notably, treatment with esomeprazole alone reduced lung collagen levels by 35% compared to the TGFβ-treated controls. Similarly, treatment with pirfenidone alone reduced lung collagen levels by 12% compared to the control group. Strikingly, the combination of pirfenidone and esomeprazole diminished lung collagen content by 57% compared to the TGFβ-treated controls that did not receive any interventional pharmacotherapy (Fig. 8). Importantly, the reduction in the level of collagen by the combination therapy is significantly more than the reduction achieved by esomeprazole or pirfenidone as monotherapy. Intriguingly, our bioinformatics study indicated that esomeprazole and pirfenidone control a number of gene sets that belong to several biological processes that are involved in lung remodeling and may have complementary effects (Fig. 5). For example, esomeprazole was shown to upregulate lung development related genes while it downregulated extracellular matrix components and several members of the collagen family that dictate collagen biosynthesis and fibril organization (Fig. 5 and Supplementary Table S1). A recent study also reported that esomeprazole regulates the aryl hydrocarbon receptor (AhR)/Smad2/3 signaling pathway to control TGFβ-induced collagen production by dermal fibroblasts and bleomycin-induced dermal and lung fibrosis in an animal model of scleroderma^42^. By contrast to esomeprazole targeted gene networks, we found that pirfenidone targets epithelial cell differentiation related genes and several members of the keratin (KRT) family including KRT5, KRT6B, KRT14 and KRT17. Although largely non-overlapping, both esomeprazole and pirfenidone co-regulated lumican (shown as LUM in Fig. 5). Lumican is a profibrotic proteoglycan that is reported to be elevated in liver, cardiac and lung fibrosis^43–45^. Taken together, complementary inhibition of profibrotic pathways and activation of lung development related gene networks using the combination of esomeprazole and pirfenidone is expected to slow or reverse the progression of lung fibrosis. Notably, antifibrotic effect of the combination therapy was observed in aged mice that are reported to closely mimic the disease process in human IPF compared to young mice that are known to possess de novo molecular repair mechanisms and naturally reverse lung fibrosis^46–48^. In addition, studies have reported that there are sex-specific differences in the development of lung fibrosis with aged male mice showing the most severe form of the disease^46^. Accordingly, the combination of pirfenidone and esomeprazole was demonstrated to slow the progression of lung fibrosis in a model that is expected to show the most severe form of the disease. However, future studies should investigate female mice to evaluate if there are sex-specific differences in response to the combination therapy.

In conclusion, our in vitro study using IPF lung fibroblasts and the in vivo study in a mouse model of TGFβ-induced lung fibrosis demonstrate that combining esomeprazole with pirfenidone improves antifibrotic efficacy of the now standard of care antifibrotic drug pirfenidone. This is an important finding given the modest antifibrotic effect of pirfenidone in patients with IPF, and the safe use of PPIs in the IPF patient population. As current FDA-approved IPF treatments only slow the rate of disease progression, combination therapy with more than one agent will likely be needed, and the repurposing of currently available and inexpensive treatments (like PPIs) is particularly appealing. By extension, our data support prospective evaluation of pirfenidone and esomeprazole combination in a randomized controlled trial in patients with IPF. However, it should be taken into consideration that optimal antifibrotic efficacy of esomeprazole (> 50 µM) is achieved at concentrations that are higher than what is achieved in the plasma of patients who take PPIs at doses commonly prescribed for the treatment of GERD^49^. Therefore, future clinical studies should consider adjusting the doses of PPIs such as esomeprazole to achieve higher target concentrations when the intention is to control processes that are involved in pathological lung remodeling beyond reducing the acidity of the gastric juice and gastric refluxate.

## Supplementary Information

Supplementary Information.

## Data Availability

All data generated or analyzed during this study are included in this manuscript and its Supplementary Information files.
